# Functional Non-coding RNA During Embryonic Myogenesis and Postnatal Muscle Development and Disease

**DOI:** 10.3389/fcell.2021.628339

**Published:** 2021-01-28

**Authors:** Hongmei Luo, Wei Lv, Qian Tong, Jianjun Jin, Zaiyan Xu, Bo Zuo

**Affiliations:** ^1^Key Laboratory of Swine Genetics and Breeding of the Ministry of Agriculture and Rural Affairs, Huazhong Agricultural University, Wuhan, China; ^2^Key Laboratory of Agriculture Animal Genetics, Breeding and Reproduction of the Ministry of Education, Huazhong Agricultural University, Wuhan, China; ^3^Department of Basic Veterinary Medicine, College of Veterinary Medicine, Huazhong Agricultural University, Wuhan, China; ^4^The Cooperative Innovation Center for Sustainable Pig Production, Wuhan, China

**Keywords:** myogenesis, muscle disease, miRNAs, lncRNAs, circRNAs

## Abstract

Skeletal muscle is a highly heterogeneous tissue that plays a crucial role in mammalian metabolism and motion maintenance. Myogenesis is a complex biological process that includes embryonic and postnatal development, which is regulated by specific signaling pathways and transcription factors. Various non-coding RNAs (ncRNAs) account for the majority of total RNA in cells and have an important regulatory role in myogenesis. In this review, we introduced the research progress in miRNAs, circRNAs, and lncRNAs related to embryonic and postnatal muscle development. We mainly focused on ncRNAs that regulate myoblast proliferation, differentiation, and postnatal muscle development through multiple mechanisms. Finally, challenges and future perspectives related to the identification and verification of functional ncRNAs are discussed. The identification and elucidation of ncRNAs related to myogenesis will enrich the myogenic regulatory network, and the effective application of ncRNAs will enhance the function of skeletal muscle.

## Introduction

**Figure 1:**
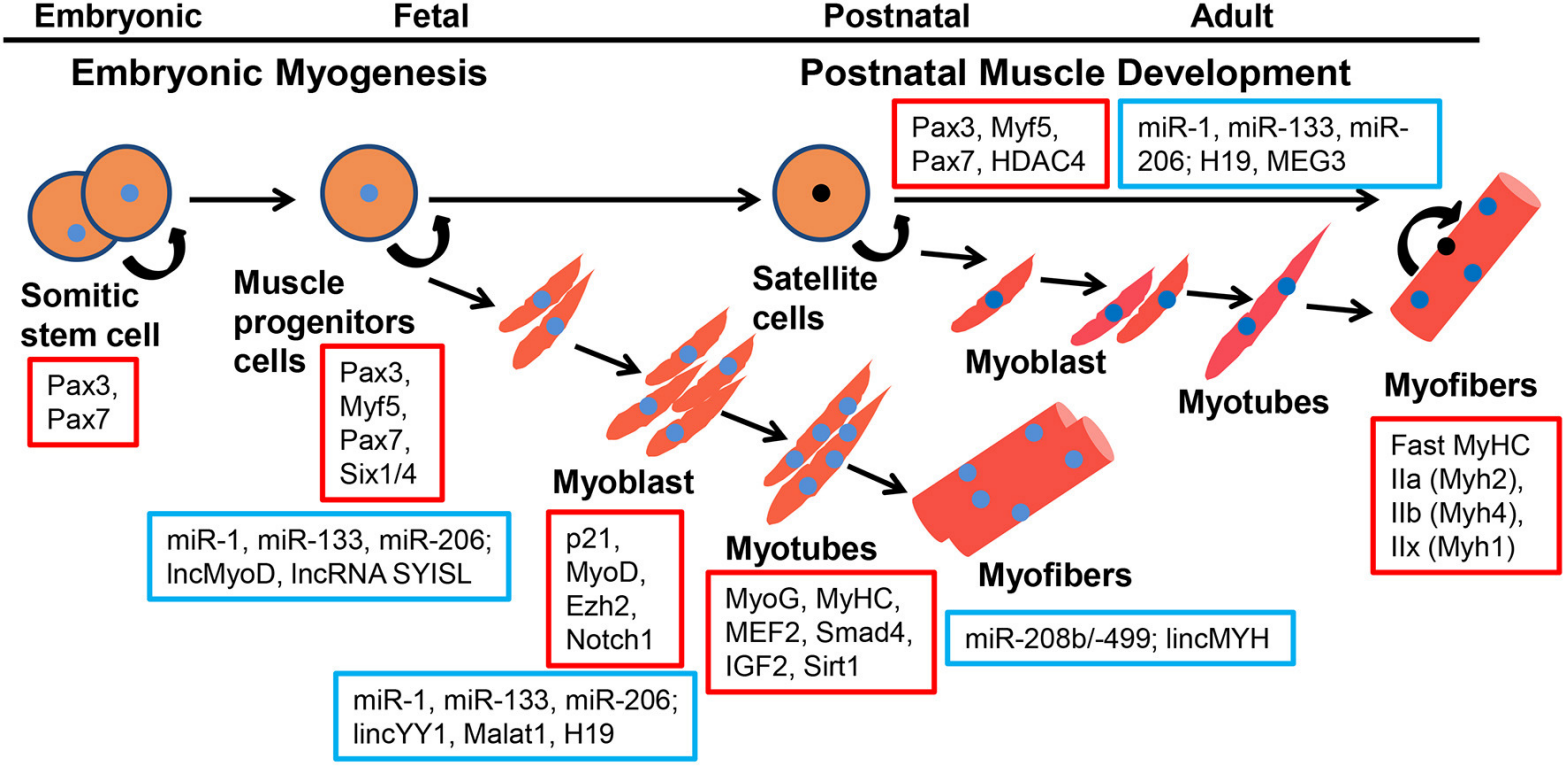
Coding genes and ncRNAs regulate the process of embyronic and postnatal myogenesis. Coding genes are represented in red squares, ncRNAs are represented in blue squares.

Skeletal muscle is a highly heterogeneous tissue that contains myofibers, the basement membrane, muscle satellite cells, immunocytes, and nerves, and plays a crucial role in locomotion, metabolism, and homeostasis. In mice and humans, this tissue represents ~30–40% of the total body mass (Zierath and Hawley, 2004). The molecular regulation of the skeletal muscle during embryonic and postnatal development is complex. Many aspects of adult myogenesis resemble embryonic morphogenetic episodes (Bentzinger et al., 2012). In vertebrate embryos, the skeletal muscles of the trunk and limbs are derived from the paraxial mesoderm and first form a certain number of somites (Bentzinger et al., 2012). Somites undergo morphogenetic changes and differentiate into sclerotome and dermomyotome, and muscle progenitor cells (MPCs) delaminate from the surrounding of the dermomyotome under the regulation of the Shh, Notch, and Wnt signaling pathways (Grefte et al., 2007). At this stage Myf5 and Mrf4, independently of Pax3/7, regulate the entry of MPCs into the myogenic program. MPCs express Pax3 and Pax7 genes, and migrate to the limbs and trunk (Buckingham and Relaix, 2007). However, some MPCs give rise to a subpopulation of postnatal muscle stem cells called satellite cells (SCs) (Gros et al., 2005). Both MPCs and SCs can give rise to myoblasts to complete myogenesis. The committed myoblast undergo proliferation, exit the cell cycle, express myogenic regulatory factors (MRFs), undergo morphological changes, fusing to form multinucleated myotubes. Finally, these myotubes are further fused into mature myofibers (Tajbakhsh, 2009) (Figure 1). Six1/4 and Pax3/7 are master regulators of MPCs but not SCs during early lineage specification, whereas Myf5 and MyoD commit MPCs and SCs to the myogenic program. MPCs and SCs expression of the terminal differentiation genes are performed by both myogenin (MyoG) and MyHC (Bentzinger et al., 2012). Skeletal muscle is composed of multinucleated contractile myofibers. Studies have shown that committed myoblasts align and fuse to generate small multinucleated myofibers during primary myogenesis in the embryo [from embryonic day 11 (E11)-E14.5]; during secondary myogenesis (from E14.5-to birth), the formation of myofibers containing hundreds of myonuclei are regulated *via* numerous signaling pathways and transcription factors (Sambasivan and Tajbakhsh, 2007).

SCs reside between the sarcolemma and basal lamina of myofibers (Mauro, 1961). SCs can serve as a pathway for skeletal muscle fiber growth after birth by activating myogenesis, repairing muscle fiber damage, or prolonging muscle fiber growth (Grounds and Yablonka-Reuveni, 1993). Under normal conditions, SCs express Pax7 and remain in a state of mitotic quiescence (Cheung and Rando, 2013). When muscles are stimulated or injured, SCs are immediately activated, proliferated, and differentiated like myoblasts. Finally, these cells fuse with the original myofibers or fuse with each other into myotubes and connect to the tail of old myofibers to form new myofibers (Zammit et al., 2006). This process is called postnatal muscle development (Relaix and Zammit, 2012). The SC population is a heterogeneous mixture of stem cells and committed progenitors (Le Grand and Rudnicki, 2007), and proliferates and divides into two daughter cells in an “asymmetric” pattern after activation (Kuang et al., 2007). Using chromosome orientation-fluorescence *in situ* hybridization in transgenic Tg:Pax7-nGFP mice, Rocheteau et al. (2012) demonstrated that all chromatids segregate asymmetrically in SCs. Cho and Doles used single cell RNA sequencing (scRNA-seq) to study the transcriptional diversity of freshly isolated skeletal muscle SCs and found the extensive transcriptional heterogeneity between individual SCs (Cho and Doles, 2017). Single-cell mass spectrometry revealed the heterogeneity of skeletal muscle SC in the activation of myogenesis *in vitro* and *in vivo* (Porpiglia et al., 2017).

Both embryonic myogenesis and postnatal skeletal muscle development is a highly regulated process; each step of the process is regulated by specific signaling pathways and transcription factors such as Sonic hedgehog (Shh), Notch, Wnt, and bone morphogenetic protein 4 (BMP4) (Jin et al., 2016), especially MRFs and post-transcriptional regulation by ncRNAs (Pauli et al., 2011; Zammit, 2017). The Encyclopedia of DNA Elements (Consortium) project showed that 80% of the eukaryotic genome is transcribed (Consortium, 2012), but <2% of total genomic sequences are transcribed into mature protein-coding RNAs, and the vast majority of transcripts are ncRNAs (Cheng et al., 2005). Various ncRNAs account for the majority of total RNA in cells. ncRNAs include tRNA, rRNA, eRNA, mitochondrial ncRNAs, micro (mi) RNAs, long non-coding (lnc) RNAs, circular (circ) RNAs, and PIWI-interacting RNAs (pi) RNAs (de Gonzalo-Calvo and Thum, 2018). Furthermore, ncRNAs are involved in diverse biological processes, and an increasing number of studies have shown that ncRNA-mediated epigenetic regulation plays an important role in myogenesis. In this review, we mainly focused on the importance of miRNAs, circRNAs, and lncRNAs in embryonic myogenesis and postnatal skeletal muscle development, and discussed their regulation of muscle disease. Finally, challenges and future perspectives in the identification of novel muscle-related ncRNAs were discussed.

## miRNAs and Myogenesis

### miRNA and Embryonic Myogenesis

miRNAs are a class of small RNAs that are 20–24 nucleotides in length, and regulate the expression of target messenger RNAs (mRNAs) through base-pairing with the 3′ untranslated regions (3′UTRs) (Bartel, 2009). This interaction leads to inhibition of translation, mRNA cleavage, and transcript degradation (Bethune et al., 2012). This mechanism reportedly guides a diverse set of RNA-induced silencing complexes (RISC) to target mRNAs (Schraivogel and Meister, 2014). The biogenesis of most miRNAs depends on specific RNA processing enzymes, including Drosha and its essential cofactor DGCR8, Dicer (Bushati and Cohen, 2007). Dicer activity is essential for normal muscle development during embryogenesis, and Dicer muscle-specific mutants reduced muscle miRNAs and led to a decrease in myofiber development by reducing muscle mass and perinatal lethality in mice (O'Rourke et al., 2007). The role of miRNAs during embryogenesis was explored in zebrafish. Giraldez and colleagues generated maternal-zygotic Dicer (MZdicer) mutants that disrupted the Dicer ribonuclease III and double-stranded RNA-binding domains. MZdicer mutants displayed abnormal morphogenesis during gastrulation, somitogenesis, and heart development. Mutant embryos failed to process precursor miRNAs into mature miRNAs, but injecting preprocessed miRNAs restored gene silencing, indicating that miRNAs are essential for early embryogenesis (Giraldez et al., 2005). A growing number of studies have elucidated that miRNAs regulate various aspects of animal embryogenesis, with some miRNAs functioning in a tissue-specific manner.

MyomiRs are a muscle-enriched group of miRNAs, mainly composed of miR-1 and miR-133 families, including miR-1/miR-1-2/miR-206 and miR133a /miR-133b (Horak et al., 2016; Mok et al., 2017). Furthermore, miR-1 and miR-133 are potent repressors of non-muscle gene expression and cell fate during mouse and human embryonic stem cell differentiation (Ivey et al., 2008). In zebrafish, downregulation of miR-1 and miR-133 altered muscle gene expression and disrupted actin organization and sarcomere assembly during muscle differentiation (Mishima et al., 2009). During embryonic myogenesis, miR-1 and miR-133 actively shaped gene expression patterns. Among the set of myomiRs, miR-206 was detected in somites of chick and mouse embryos (Sweetman et al., 2006). Sweetman et al. (2008) demonstrated that the ectopic expression of MRFs in the developing chicken neural tube induced the expression of distinct myomiRs such as miR-1 and miR-206, whereas the lack of *Myf-5* resulted in a loss of myomiR expression in developing somites. Their results indicated that myomiRs regulate myogenesis through MRFs. Additional gain- and loss-of-function experiments are needed to illustrate the role of myomiRs during myogenesis by affecting MRF expression. In *Xenopus laevis*, Vergara et al. examined the expression of miR-206 accompanying somitogenesis. Both knockdown and overexpression of miR-206 resulted in abnormal somite formation (Vergara et al., 2018). Conversely, miR-133 knockdown impaired myotome formation and growth, and the evolutionarily conserved miR-133 family mediated Gli3 silencing was critical for embryonic myogenesis (Mok et al., 2018). Other studies have specifically focused on the genetic analysis of myomiRs in a variety of model organisms, including flies, zebrafish, and mice (Sokol, 2012). MyomiRs are integrated into myogenic regulatory networks and, in turn, have widespread control of muscle gene expression.

In addition to myomiRs, several other miRNAs are involved in skeletal muscle embryonic development. For example, miR-196 reportedly acts upstream of Hoxb8 and Shh *in vivo* in the context of limb development, and primarily regulated the transcription of myogenesis (Hornstein et al., 2005). The zebrafish *Myf5* locus has an intronic miRNA, termed miR-3906 or miR-In300, which was found to impair fast muscle differentiation by targeting *Homer-1b* or *Dmrt2a*, respectively, in zebrafish embryos (Lin et al., 2013). miR-203 was transiently upregulated in chicken embryos on days 10 to 16 (E10–E16)c and was sharply downregulated and not expressed after E16 in the chicken embryonic skeletal muscle. Histological profiles and weight variations of embryo skeletal muscle revealed that miR-203 expression correlates with muscle embryonic development (Luo et al., 2014). Collectively, these results demonstrated the expression and function of myomiRs in skeletal embryonic myogenesis.

### Functional Analysis of miRNAs During Postnatal Muscle Development

In recent years, a fraction of miRNAs has been detected in the skeletal muscle. Herein, we systematically summarized the functions and regulatory mechanisms of miRNAs (Table 1) during postnatal skeletal muscle development.

**Table 1:** Functions and regulatory mechanisms of miRNAs in postnatal skeletal muscle development.

**miRNA**	**Functions**	**Mechanisms**	**Species**	**References**
DmiR-1	Required for the dramatic post-mitotic growth of larval muscle	Targets twist	*Drosophila*	Sokol and Ambros, 2005
miR-1	Promotes myoblast proliferation and differentiation	Targets HDAC4	Mouse	Chen et al., 2006
	Maintains SCs quiescence and promotes self-renewal		Goat	Sui et al., 2020
miR-181	Required for skeletal myoblast terminal differentiation	Targets HoxA11	Mouse	Naguibneva et al., 2006
miR-214	Modulation of Hedgehog (Hh) signaling in somite cells, enhance slow-muscle cell types	Targets suppressor of fused [su(fu)]	Zebrafish	Flynt et al., 2007
	Promotes myoblast differentiation	Targets Ezh2	Mouse	Juan et al., 2009
miR-221/222	Inhibits proliferation and differentiation	Targets p27	Quail	Cardinali et al., 2009
miR-208b/-499	Activates slow and represses fast myofibers gene programs	Targets Sox6	Mouse	van Rooij et al., 2009
miR-27b	Promotes myoblast differentiation	Targets Pax3	Mouse	Crist et al., 2009
	Promotes myoblast proliferation and differentiation		Goat	Ling et al., 2018
	Promotes myoblast differentiation	Inhibits MDFI expression	Porcine	Hou et al., 2018
miR-206	Promotes myoblasts proliferation and differentiation	Targets Pax7	Mouse	Chen et al., 2010
miR-486	Promotes myoblast differentiation	Targets Pax7	Mouse	Dey et al., 2011
miR-155	Inhibits myoblast differentiation	Targets MEF2A	Mouse	Seok et al., 2011
miR-125b	Inhibits myoblast differentiation	Targets IGF2	Mouse	Ge et al., 2011
miR-26a	Promotes myoblast differentiation	Targets Smad1 and Smad4	Mouse	Dey et al., 2012
miR-489	Maintains SCs quiescence and regulates self-renewal	Post-transcriptionally suppresses the oncogene Dek	Mouse	Cheung et al., 2012
miR-31	Maintains SC quiescence	Sequesteres in mRNP granules together with Myf5 mRNAs.	Mouse	Crist et al., 2012
miR-23a	Inhibits myogenic differentiation	Targets fast myosin heavy chain (Myh) genes, including Myh 1, 2 and 4	Mouse	Cornu et al., 2012
miR-128a	Negatively regulates myoblast proliferation and myotubes hypertrophy	Regulates IRS1/Akt insulin signaling	Mouse	Motohashi et al., 2013
miR-675-3p/-5p	Promotes myoblast differentiation and regeneration	Targets Smad transcription factors	Mouse	Dey et al., 2014
miR-146b	Promotes myogenic differentiation	Targets Smad4, Notch1, and Hmga2	Mouse	Khanna et al., 2014
miR-195/497	Maintains SCs quiescence	Targets Ccnd1 and Ccnd2	Mouse	Sato et al., 2014
miR-186	Inhibits myoblast differentiation	Targets myogenin	Mouse	Antoniou et al., 2014
miR-30a	Promotes myogenesis by increasing apoptosis and altering somite morphology	Targets Six1	Zebrafish	O'Brien et al., 2014
miR-431	Promotes differentiation and regeneration of old skeletal muscle	Targets Smad4	Mouse and human	Lee et al., 2015
miR-15b/miR-23b/miR-106b/miR-503	Pitx2-miRNA pathway regulates myoblast proliferation	Targets CyclinD1 and CyclinD2	Mouse	Lozano-Velasco et al., 2015
miR-20a/20b	Promotes myoblast differentiation and represses myoblast proliferation	Targets E2F transcription factor 1 (E2F1)	Chicken	Luo et al., 2016
miR-29a	Promotes SCs proliferation	Targets FGF2	Mouse	Galimov et al., 2016
miR-17-92	Promotes myoblast proliferation but inhibits myotubes formation	Targets ENH1	Mouse	Qiu et al., 2016
miR-34c	Inhibits myoblast proliferation and promotes differentiation	A regulatory loop with Notch1	Mouse	Hou et al., 2017
miR-139	Promotes SCs differentiation	Targets DHFR	Bovine	Zhou et al., 2018
miR-708	Activates SCs and regulates self-renewal	Antagonizes Tensin3 to inhibit FAK activation	Mouse	Baghdadi et al., 2018
miR-487b-3p	Suppresses the proliferation and differentiation of myoblast	Targets IRS1	Goat	Wang et al., 2018
miR-17/19	Promotes myoblast differentiation	miR-17 targets Ccnd2, Jak1 and Rhoc, miR-19 complement miR-17	Mouse	Kong et al., 2019
miR-208b	Promotes myoblast proliferation, inhibits differentiation	Targets the E-protein family member TCF12	Mouse	Fu et al., 2020
	Stimulates fast-to-slow fiber conversion and oxidative metabolism programmers	Targets FNIP1		
miR-9-5p	Inhibits the proliferation and differentiation of SCs	Targets IGF2BP3	Chicken	Yin et al., 2020

Several 100 miRNAs have been identified in plants, animals, and viruses by employing molecular cloning and bioinformatic approaches. In different tissues, miRNAs were found to downregulate gene expression by base-pairing with the 3′UTRs of target mRNAs; they also function this mechanism during myogenesis. For instance, miRNAs can influence proliferation *via* mRNAs coding regulators of the cell cycle. miR-27b is a functionally conserved miRNA that targets Pax3 to promote myoblast proliferation in mouse and goat (Crist et al., 2009; Ling et al., 2018). miR-195/497 is a positive regulator of SC quiescence by targeting Ccnd1 and Ccnd2 (Sato et al., 2014). The insulin-like growth factor (IGF) pathway, myocyte enhancer factor-2 (MEF2), and MRF factors play vital roles in myogenesis, miRNAs can regulate these pathways. For example, IGF-2, a critical regulator of skeletal myogenesis, is a direct and major target of miR-125b in both myocytes and regenerating muscles. miR-125b negatively modulated myoblast differentiation *in vitro* and muscle regeneration *in vivo* (Ge et al., 2011). MEF2A is a member of the MEF2 family of transcription factors, and miR-155 significantly suppressed the expression of MEF2A, repressing skeletal muscle differentiation (Seok et al., 2011). MRFs, including Myf5, MyoD, Myf6, and myogenin, regulate skeletal muscle differentiation, where myogenin plays a critical role in regulating the final stage of muscle differentiation. Antoniou et al. (2014) predicted that six miRNAs (miR-182, miR-186, miR-135, miR-491, miR-329, and miR-96) bind the myogenin 3′-UTR, but only miR-186 is a novel post-transcriptional regulator of myogenin during skeletal myogenesis. Recently, Fu et al. showed that miR-208b targeted the E-protein family member TCF12 to promote mouse myoblast proliferation and inhibit their differentiation. Meanwhile, miR-208b stimulated fast-to-slow fiber conversion and oxidative metabolism process by targeting FNIP1, thereby regulating postnatal muscle development (Fu et al., 2020). Several miRNAs are reportedly present in muscle cells and their modulating influence on myogenesis is likely to be overly complex. Some miRNAs target the same transcriptional networks in different species (Supplementary Table 1).

## cicrRNA During Myogenesis

### cicrRNA and Embryonic Myogenesis

With the advent of high-throughput sequencing and novel bioinformatic tools, thousands of circRNAs were discovered and their abundance and function were recorded (Salzman et al., 2012; Memczak et al., 2013). circRNAs are characterized by a covalently closed ring structure without 3′ and 5′ ends and are generated by precursor mRNA back-splicing of exons. Typically, the expression levels of circRNAs are low, often exhibiting cell- and tissue-specific patterns in eukaryotes (Li et al., 2018c). Increasing evidence has demonstrated that circRNAs participate in many steps of gene expression by acting as miRNA sponges, miRNA decoy, RNA-binding proteins (RBPs), as well as mediating RNA translation and protein interaction. Several studies have demonstrated that circRNAs possess coding capabilities (Panda et al., 2017). Owing to the non-linear conformation of circRNAs and lack of polyadenylated [poly (A)] tails, very few circRNAs can be identified by RNA-seq, but circRNAs are readily detectable in ribosomal RNA-depleted RNA-seq datasets (Greco et al., 2018). One microarray analysis found that 581 circRNAs were differentially regulated between C2C12 myoblasts and myotubes (Chen et al., 2018a). However, their roles remain to be explored. Notably, circRNAs may play a vital role in myogenesis. Fan et al. (2015) developed a single-cell universal poly(A)-independent RNA sequencing (SUPeR-seq) method to sequence both polyadenylated and non-polyadenylated RNAs from individual cells, discovering 2,891 circRNAs and 913 novel linear transcripts in mouse preimplantation embryos. This research is crucial to decipher functional regulators of circRNAs during early mammalian embryonic development. An increasing number of databases have suggested that skeletal muscle is one of the tissues enriched in circRNAs (Cheng et al., 2005; Li et al., 2017).

circSVIL, an exonic circular, was differentially expressed in chicken skeletal muscle at E11, E16, and post-hatching day 1 (P1) (Ouyang et al., 2018b). circSVIL functions as miR-203 sponges and upregulates levels of *c-JUN* and *MEF2C*, thereby promoting the proliferation and differentiation of myoblasts (Ouyang et al., 2018a). circFGFR2, generated by exon 3–6 of the *FGFR2* gene, was differentially expressed during chicken embryo skeletal muscle development (Chen et al., 2018b). circFGFR2 directly targeted miR-133a-5p and miR-29b-1-5p and further eliminated the inhibitory effects of the two miRNAs on myoblast proliferation and differentiation (Chen et al., 2018b). The circRNA sequencing data of bovine skeletal muscle tissue demonstrated that circFUT10 was highly (but differentially) expressed in embryonic and adult skeletal muscle tissues. Reportedly, circFUT10 regulated myoblast differentiation and cell survival by directly binding to miR-133a and inhibiting miR-133a activity (Li et al., 2018b). circFUT10 may target myomiRs to regulate embryonic myogenesis. The expression level of circSNX29 was considerably higher in the bovine embryonic skeletal muscle than in adult skeletal muscle. circSNX29 directly interacted with miR-744 and efficiently reversed the suppression of Wnt5a and calcium/calmodulin–dependent protein kinase II delta (CaMKIIδ) (Peng et al., 2019). In bovine, enhancing circFUT10 or circSNX29 expression may emerge as a potential target in breeding strategies attempting to control muscle development. Overall, circRNAs play a role in regulating the myoblast cycle and development by acting as miRNA binding sites to facilitate the regulation of gene expression during myogenesis (Zhang et al., 2018a). In Duroc pigs, Hong et al. (2019) performed RNase R+RNA-seq in three distinct stages of embryonic skeletal muscle development (33, 65, and 90 days prenatal) to identify circRNAs and found that many circRNAs were specifically expressed at different embryonic stages. Collectively, these findings are helpful for further research on circRNAs in myogenesis.

### circRNAs Regulating Postnatal Muscle Development

Here, we summarized the current research progress on the role of circRNAs in postnatal myogenesis (Table 2).

**Table 2:** Functions and regulatory mechanisms of circRNAs in skeletal muscle development.

**circRNA**	**Functions**	**Mechanisms**	**Species**	**References**
circLMO7	Inhibits myoblast differentiation and promotes myogenesis	miR-378a-3p sponge	Bovine	Wei et al., 2016
circ-ZNF609	Promotes myoblast proliferation	Protein encoding	Human	Legnini et al., 2017
circRBFOX2	Promotes myoblast proliferation	mir-206 sponge	Chicken	Ouyang et al., 2018b
circFGFR4	Promotes myoblast differentiation and apoptosis	miR-107 sponge	Bovine	Li et al., 2018a
circ-Zfp609	Inhibits myoblast differentiation	miR-194-5p sponge	Mouse	Wang et al., 2019b
circHIPK3	Promotes myoblast proliferation and myogenesis	miR-30a-3p sponge	Chicken	Chen et al., 2019
	Promotes myoblast differentiation	miR-124 and miR-379 sopnge	Mouse	Yao et al., 2020
circ-FoxO3	Inhibits myoblast differentiation	miR-138-5p sponge	Mouse	Li et al., 2019b
circTitin (circTTN)	Promotes proliferation and differentiation of bovine primary myoblast	miR-432 sponge	Bovine	Wang et al., 2019a
circTMTC1	Inhibits chicken SMSC differentiation	miR-128-3p sponge	Chicken	Shen et al., 2019
CDR1as (ciRS-7)	Promotes myogenic differentiation	miR-7 sponge	Goat	Li et al., 2019a
circHUWE1	Facilitates myoblast proliferation, inhibits apoptosis and differentiation	miR-29b sponge	Bovine	Yue et al., 2020a
circSamd4	Promotes myogenic differentiation	Associates with PUR proteins	Mouse	Pandey et al., 2020
circINSR	Promotes proliferation and reduces apoptosis of bovine embryonic myoblast	miR-34a sponge	Bovine	Shen et al., 2020

Interestingly, almost all circRNAs can act as miRNA sponges to regulate the transcription and splicing of target genes. For example, circHIPK3 promoted the proliferation and differentiation of chicken myoblast cells by sponging miR-30a-3p binding to MEF2C (Chen et al., 2019). circHIPK3 can promote the differentiation of C2C12 myoblasts as a sponge of miR-124 and miR-379 (Yao et al., 2020). circINSR promoted proliferation and reduced apoptosis of bovine embryonic myoblasts by sponging miR-34a (Shen et al., 2020). Some circRNAs can be translated into proteins in the post-transcriptional regulation of muscle development. circ-ZNF609 specifically regulated mouse and human myoblast proliferation. circ-ZNF609 can be translated into a protein in a splicing-dependent and cap-independent manner when ectopically expressed (Legnini et al., 2017). circZfp609, the mouse homolog of circ-ZNF609, can sponge miR-194-5p to sequester its inhibition on BCLAF1 to repress myogenic differentiation (Wang et al., 2019b). Recently, researchers have shown that circSamd4, which is conserved between humans and mice, has a positive function in skeletal muscle differentiation by associating with PURA and PURB, two repressors of myogenesis that inhibit transcription of the myosin heavy chain (MHC) protein family (Pandey et al., 2020). This illustrated the protein interaction mechanism of circRNAs.

## lncRNAs in Myogenesis

### Role of lncRNAs in Regulating Embryonic Myogenesis

lncRNAs were originally considered as genomic transcription “noise” and account for a large proportion of total ncRNAs (Kapranov et al., 2007; Struhl, 2007). With weak or no protein-coding potential, lncRNAs are a class of RNA more than 200 nucleotides in length, possessing complex spatial structures and diverse functions (Derrien et al., 2012). Several lncRNAs are transcribed by RNA polymerase II (Pol II) from genomic loci with similar chromatin states to mRNAs; they are often 5′-capped, spliced, polyadenylated, with the absence of a translated open reading frame (ORF) (Quinn and Chang, 2016). lncRNAs are ubiquitous in organisms and are cell-type-specific, with poor evolutionary conservation among different species (Engreitz et al., 2016). Numerous studies have shown that lncRNAs are involved in the regulation of gene expression, epigenetics, cell differentiation, apoptosis, metabolism, signal transduction, and immune response (Mattick, 2005; Zhang et al., 2019). Interestingly, emerging studies have also demonstrated that some lncRNAs can encode micropeptides shorter than 100 amino acids to exert micropeptide-mediated functions (Anderson et al., 2015). Many computational pipelines developed from poly (A) RNA-seq in different cells can identify new lncRNAs, and several studies have shown that lncRNAs participate in embryo myogenesis.

H19 was one of the earliest known examples of imprinted lncRNAs that did not contain any conserved ORFs between mice and humans (Brannan et al., 1990). H19 is strongly repressed after birth in all mouse tissues, but it remains expressed in the skeletal muscle and heart in adults, suggesting an important function in these muscles (Poirier et al., 1991). Gabory and colleagues found that the H19 gene participates as a *trans* regulator in the fine-tuning of this imprinted gene network (IGN) in the mouse embryo (Gabory et al., 2009). This is the first *in vivo* evidence of a functional role for H19. Pauli et al. (2012) performed a time-series of RNA-seq experiments at eight stages during early zebrafish embryo development and observed that lncRNAs were particularly numerous during very early embryo development. *In situ* hybridization showed that lncRNAs are expressed in narrower time windows and are specifically enriched in early-stage embryos. Whole-mount *in situ* hybridization showed that lncIRS1 was expressed in the forming somites in the HH10 chick embryo, controlled IRS1 protein levels, and further activated the IGF-1 signaling pathway by functioning as ceRNA to sponge miR-15a, miR-15b-5p, and miR-15c-5p (Li et al., 2019c). Regarding lncRNAs in embryonic myogenesis, a review by Bouckenheimer et al. summarized their expression patterns and roles during early human embryo development and in pluripotent stem cells (PSCs). Importantly, abundant public mRNA sequencing (mRNA-seq) data have been used to illustrate the large number of lncRNAs expressed during embryo development (Bouckenheimer et al., 2016). These results support the notion that lncRNAs are part of dynamic changes in transcript expression occurring during mammalian early embryo development, including myogenesis. Sweta et al. suggested that lncRNAs are important for mesodermal specification and further differentiation, development, and functions of mesodermal derivatives, including *lncRNA Evx1as, lncRNA HoxBlinc, and yylncT* (Sweta et al., 2019). During embryonic development, the musculature of the adult body is derived from the mesoderm. Accordingly, these lncRNAs may function early myogenesis. However, insufficient evidence is available. Collectively, a better understanding of lncRNAs that can regulate the development of skeletal muscle will open potential avenues for their efficacious production, and enhance our knowledge regarding embryonic myogenesis development.

### Functions and Mechanisms of lncRNAs in Postnatal Muscle Development

**Figure 2:**
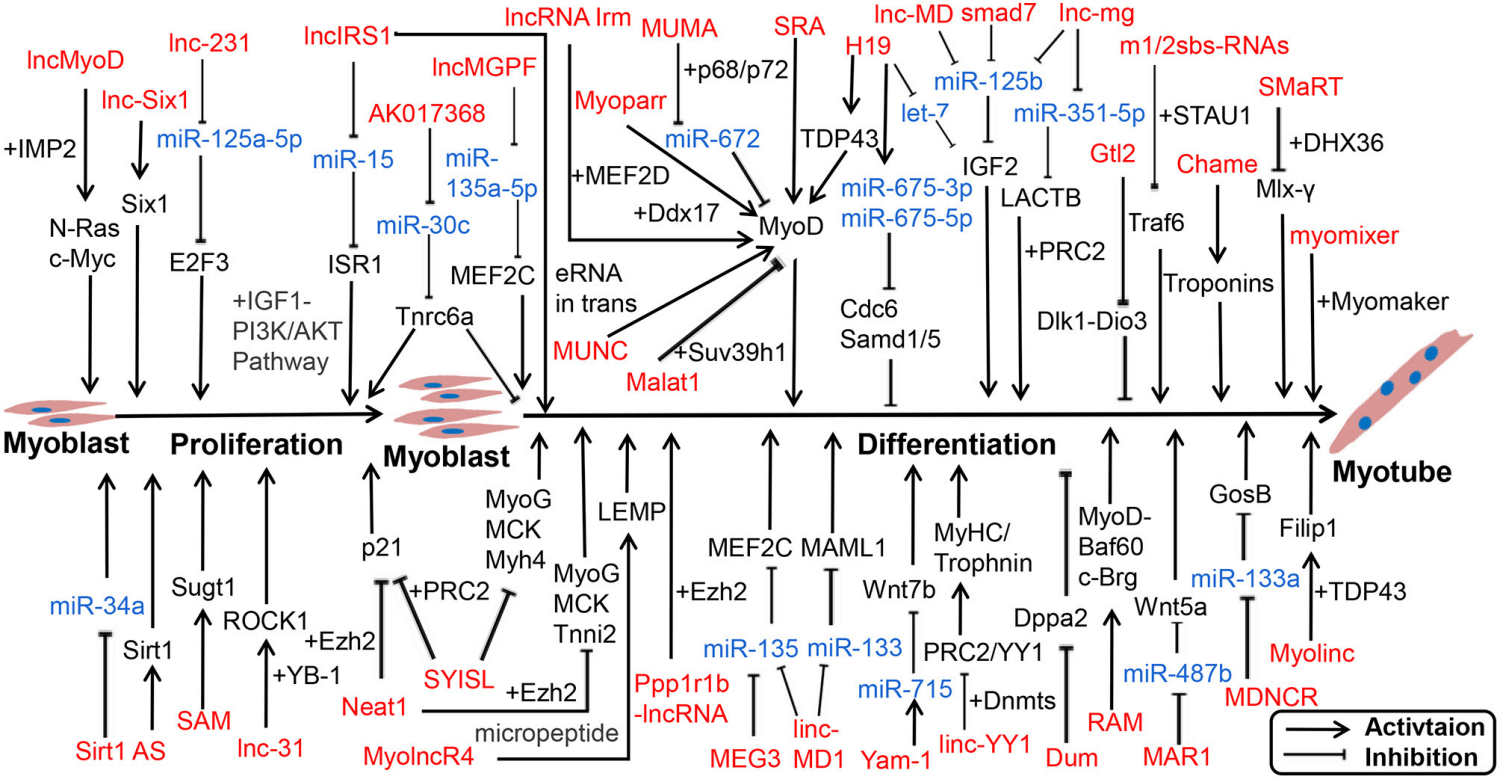
lncRNA-miRNA-gene network regulates proliferation and differentiation of myoblast. lncRNAs are represented in red, miRNAs are represented in blue.

Numerous lncRNAs have been detected in skeletal muscle, but only a small fraction of lncRNAs have been characterized. Recent reports have indicated lncRNAs exert functional roles through multiple mechanisms, including transcription activation, molecular sponge activity, competitive binding, mRNA translation, and protein stability. Studies have shown that numerous lncRNAs interact with miRNAs to facilitate myogenesis. We displayed a known pattern of lncRNAs during skeletal muscle development (Figure 2; Supplementary Table 2).

Notably, functions of lncRNAs are associated with their subcellular location and microenvironment (Chen, 2016). linc-YY1 activated gene expression *in trans* by interacting with YY1 and removing the YY1/PRC2 complex from target promoters such as Myh and Troponin, thus promoting muscle differentiation and regeneration (Zhou et al., 2015). SYISL interacted with Ezh2, the core component of PRC2, to regulate the expression of p21 and muscle-specific genes such as MyoG, MCK, and Myh4, leading to the promotion of myoblast proliferation and inhibition of myogenic differentiation (Jin et al., 2018). In the nucleus, lncRNAs can also bind to transcription factors or RBPs to influence transcription activation. For instance, lncRNA SRA promoted differentiation as a coregulator of MyoD, along with RNA helicase p68/p72 (Caretti et al., 2006; Hube et al., 2011). linc-RAM, which is induced by MyoD, interacts with MyoD, and supports the assembly of the MyoD-Baf60c-Brg complex to promote muscle growth and regeneration (Yu et al., 2017).

Most lncRNAs in the cytoplasm act as ceRNAs with miRNAs, such as lncMD1 (Cesana et al., 2011; Legnini et al., 2014), lnc-mg (Zhu et al., 2017; Du et al., 2019), and lnc-MAR1 (Zhang et al., 2018b). Recently, Li reported that 2310043L19Rik (lnc-231) inhibited differentiation and promoted proliferation of myoblasts as ceRNA to target miR-125a-5p, thereby inhibiting the function of E2F3 mRNA (Li et al., 2020b). Conversely, H19 can act as a molecular scaffold to recruit TDP43 to promoters of MyoD and activate transcription, thereby promoting porcine SC differentiation (Li et al., 2020a). Furthermore, some lncRNAs can give rise to functional micropeptides. A conserved lncRNA, LINC00961/5430416O09Rik, which is localized on the late endosome/lysosome and encodes a polypeptide of 90 amino acids termed small regulatory polypeptide of amino acid response (SPAR), interacted with lysosomal v-ATPase to negatively regulate mammalian target of rapamycin complex 1 (mTORC1) activation, as well as skeletal muscle regeneration (Matsumoto et al., 2017; Tajbakhsh, 2017). Myoregulin (MLN) and dwarf open reading frame (DWORF) are micropeptide-encoded tissue-specific putative lncRNAs, located at the sarcoplasmic reticulum membrane (Ivey et al.), and can directly bind SERCA; MLN inhibits SERCA activity and hinders the uptake of Ca^2+^ into the SR. However, DWORF increases SERCA activity by reducing exercise performance and Ca^2+^ uptake into the SR (Anderson et al., 2015; Nelson et al., 2016). As the sequence length of lncRNAs is larger than that of miRNAs and circRNAs, they function diverse mechanisms and play extensive roles in life processes.

Previous studies have focused on the function of ncRNAs in the nucleus and cytoplasm. Mitochondria are important organelles and the main energy metabolism sites in cells (Nunnari and Suomalainen, 2012). The roles of ncRNAs in mitochondria have become a new biology research topic (Bandiera et al., 2011). Barrey et al. for the first time demonstrated the presence of pre-miRNA and miRNA in the human mitochondria isolated from skeletal muscular cells (Barrey et al., 2011). The muscle-specific miR-1 is able to stimulate mitochondrial translation of multiple mtDNA-encoded transcripts, while repressing its nuclear DNA-encoded targets in the cytoplasm (Zhang et al., 2014). Some lncRNAs generated from the mammalian mitochondrial genome or located in mitochondria have been identified. Rackham et al. (2011) identified three lncRNAs generated from the mitochondrial genome named lncND5 RNA, lncND6 RNA and lncCytb RNA. Ro et al. reported that the mouse and human mitochondrial genomes encodes abundant small RNAs and named mitochondrial genome-encoded small RNAs (mitosRNAs), which may play an important regulatory role in the control of mitochondrial gene expression (Ro et al., 2013). The regulation mechanism and function of mitochondrial ncRNAs in myogenesis remain to be further explored.

As mentioned above, ncRNAs play an important role in regulating myogenesis during embryonic and adult stages. Crosstalk between miRNA-circRNA-lncRNA appears to be common in muscle development. lncRNAs can inhibit the function of miRNA through sequence-specific binding, whereas circRNAs can act as molecular sponges for miRNAs to regulate target mRNAs related to myogenesis. Further insights into postnatal skeletal muscle development will illustrate complex and dynamic regulatory networks.

## ncRNAs in Muscle Disease

**Figure 3:**
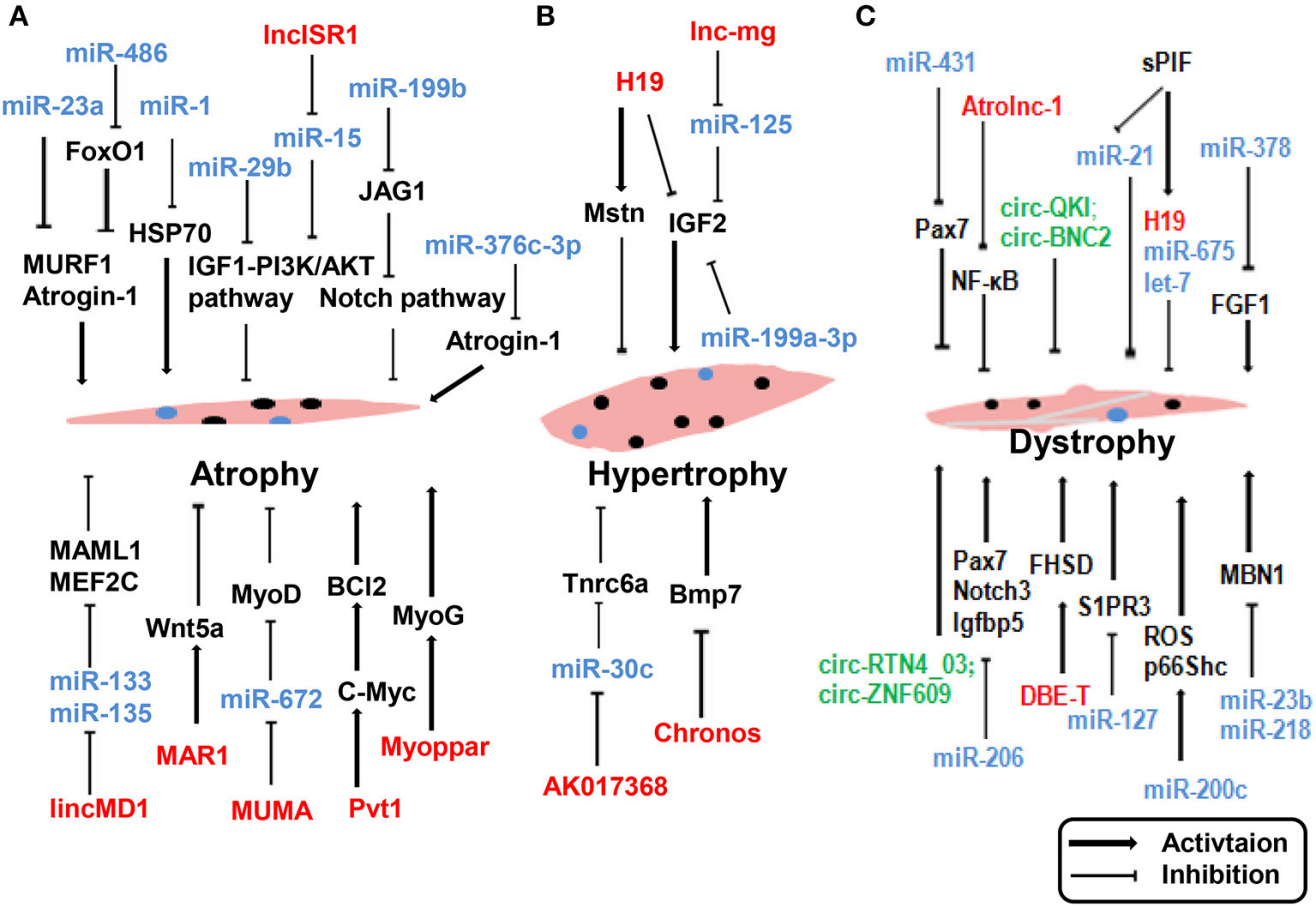
ncRNAs regulate muscle atrophy **(A)**, hypertrophy **(B)**, and dystrophy **(C)**. lncRNAs are represented in red, miRNAs are represented in blue, circRNAs are represented in green. Myoblast, black nuclei; satlliate cells, blue nuclei.

Skeletal muscle is a complex tissue in mammals, and skeletal muscle diseases are known to occur owing to physiological and pathological factors. Common skeletal muscle diseases include atrophy (Cohen et al., 2015), Duchenne muscular dystrophy (DMD) (Matsumura et al., 1994), and hypertrophy (Walters, 2017). Several reports have suggested that ncRNAs may play a functional role in muscle disease and could be potentially exploited as therapeutic tools. The impact of ncRNA dysregulation in muscle disease reported in recent years is depicted in Figure 3.

An understanding of how ncRNAs regulate skeletal muscle functions and disease can provide novel therapeutic targets for the prevention and treatment of muscle pathologies in metabolic diseases. Two muscle-specific ubiquitin ligases, MAFbx/Atrogin-1 and muscle RING-finger 1 (MuRF1), are prominently induced during muscle atrophy and mediate atrophy-associated protein degradation (Sacheck et al., 2007). Blocking the expression of these two ubiquitin ligases affords protection against muscle atrophy. Wada and colleagues reported that miR-23a suppressed the translation of both MAFbx/Atrogin-1 and MuRF1 in a 3′-UTR-dependent manner. Ectopic expression of miR-23a was sufficient to protect the muscle from atrophy both *in vitro* and *in vivo* (Wada et al., 2011). Direct interactions were identified between miR-376c-3p and the 3′ UTR of Atrogin-1, leading to repression of Atrogin-1, and thereby induction of eIF3f protein levels in both human and mouse skeletal muscle cells (Shin et al., 2020). Reportedly, lncRNA Pvt1 is upregulated during muscle atrophy by blocking c-Myc phosphorylation and degradation. Functionally, Pvt1 affects mitochondrial respiration and morphology, thereby affecting muscle atrophy and myofiber size *in vivo* (Alessio et al., 2019). In addition to affecting myogenesis during embryonic and postnatal development, muscle disease is affected by miR-206. Loss of miR-206 accelerated and exacerbated the dystrophic phenotype in mdx mice by suppressing Pax7, Notch3, and Igfbp5 (Liu et al., 2012). In contrast, several lncRNAs are known to affect the pathological status of skeletal muscle dystrophy. lncRNA Atrolnc-1 was abundantly expressed in skeletal muscle and markedly increased in atrophying muscle. Atrolnc-1 strongly binds to ABIN-1, inhibits NF-κB signaling, and causes protein degradation in muscle cells (Sun et al., 2018). H19 can influence muscle hypertrophy by regulating the expression of Mstn and IGF2 (Martinet et al., 2016). Neppl and colleagues observed that lncRNA Chronos was expressed in muscles. Inhibition of Chronos induced myofiber hypertrophy both *in vitro* and *in vivo* through the epigenetic modulation of Bmp7 signaling (Neppl et al., 2017). Knockdown of lncRNA AK017368 promoted muscle hypertrophy *in vivo*. Notably, lncRNA AK017368 promoted proliferation and inhibited differentiation of myoblasts by competing with Tnrc6a for miR-30c (Liang et al., 2018).

Furthermore, circRNAs are associated with muscle disease. The dystrophy gene was among the first genes identified to generate circRNAs in skeletal muscle (Surono et al., 1999). Legnini et al. analyzed data from both normal and dystrophic human myoblasts and identified circ-QKI and circ-BNC2 upregulated during *in vitro* differentiation, and downregulated in the DMD conditions, which is consistent with the notion that dystrophic cells have altered progression into the differentiation process (Legnini et al., 2017). Song et al. identified 197 differentially expressed circRNAs between mdx mice and C57 mice by microarray analysis. A circRNA/miRNA interaction network was predicted by bioinformatic approaches (Song et al., 2020). These studies described the expression pattern of circRNAs and indicated that circRNAs may play pivotal roles in the pathophysiological mechanisms of DMD.

## Conclusion and Future perspective

The discovery of ncRNAs has remarkably broadened our understanding of epigenetic regulation. Most researchers have used overexpression or inhibition of ncRNA function to explore their mechanisms during myogenesis; moreover, some ncRNAs have been well-investigated using *in vivo* mouse models. Several reports have revealed ncRNA functions in skeletal muscle development and disease and can be developed as novel biomarkers or targets for improving muscular abilities and therapeutic strategies.

Despite improvements in our knowledge regarding the role of ncRNAs in myogenesis, detailed identification and verification of ncRNAs still encounter several obstacles, including the following:

1. several techniques have been used to identify ncRNAs, including ncRNA microarray analysis and sequencing methods based on poly (A) sequencing, especially next-generation sequencing (NGS). The limitations of NGS on the identification of ncRNAs and its regulatory mechanisms need to be addressed as non-poly (A) forms of ncRNAs such as circRNAs are often ignored. Furthermore, there are a variety of ncRNA databases available, such as miRBase, TargetScan, ribosomal RNA-depleted RNA-seq datasets, lncRNA Database v2.0, and Linc2GO. We need to determine a systematic identification approach to combine ncRNA data generated from different methodologies.
2. Most known miRNAs and circRNAs demonstrate cytoplasmic localization, whereas lncRNAs are present in both the nucleoli and cytoplasm. Furthermore, nuclear miRNA-directed gene regulation constitutes a departure from the prevailing view of miRNA functions (Roberts, 2014), which requires different experimental schemes to identify functional ncRNAs. Fang et al. developed PIRCh-seq, a method that enables a comprehensive survey of chromatin-associated ncRNAs in a histone modification-specific manner (Fang et al., 2019). PIRCh-seq significantly reduces the influence of nascent transcripts and more precisely reveals relationships between chromatin and its associated ncRNAs than other sequencing methods. More recently, ribosome profiling, droplet digital PCR, and NanoString Technologies nCounter assays have uncovered the functions of ncRNAs (Chen, 2020).
3. Current experiments need to be based on the million-cell scale, and their accuracy does not support the single-cell level experiment. The current omics research field has been transformed from mixed sample research to single-cell level research. Single-cell sequencing technology has revealed the existence of cellular heterogeneity and related molecular mechanisms in detail. For some precious cell samples or embryonic samples, rigorous validation experiments are needed.
4. The mechanism of ncRNAs has been assessed *via* RIP, ChIRP, CHART, or GRID. One limitation of these probe approaches is that their efficacy is based on the size and location of ncRNAs. In recent years, many studies have reported that ncRNAs play biological functions through exosomes mediating intercellular communication (Romancino et al., 2013). Exosomes have been described as 40–100 nm vesicles that are secreted by a broad range of cell types and have been identified in diverse body fluids (Raposo and Stoorvogel, 2013). Exosomes have classically double membrane structure containing rich source of proteins, lipids, mRNAs and miRNA biological ingredients (Chevillet et al., 2014). The proteomic analysis of C2C12 myoblast and myotube exosome-like vesicles showed that exosomes could regulate muscle development (Forterre et al., 2014b). Myotube-derived exosomal miRNAs downregulate Sirtuin1 in myoblasts during muscle cell differentiation (Forterre et al., 2014a). Hudson et al. found that the expression of miR-23a in exosomes was altered, which weakened its inhibitory effect on target genes MuRf1 and atrogin-1, and leaded to muscle atrophy (Hudson et al., 2014). Mesenchymal-stem-cell-derived exosomes had low concentrations of muscle-repair-related cytokines and a number of repair-related miRNAs such as miR-494 to promote myogenesis and angiogenesis *in vitro* (Nakamura et al., 2015). There is growing evidence indicating active function of lncRNAs and circRNAs in exosomes (Yue et al., 2020b). Bioactive lncARSR (lncRNA Activated in RCC with Sunitinib Resistance) could be incorporated into exosomes and transmitted to sensitive cells, thus disseminating sunitinib resistance (Qu et al., 2016). H19 can be transferred from carcinoma-associated fibroblasts (CAFs) to colorectal cancer cells (CRCs) through exosomes, and acts as a competing endogenous RNA sponge for miR-141 in CRCs, promoting the stemness and chemoresistance of CRCs (Ren et al., 2018). However, the ncRNAs of exosomes related to myogenesis needs more in-depth analysis.
5. The functional interaction maps for a majority of characteristic ncRNAs with DNA or transcription factors, except for some well-studied cases, remain largely unknown. One crucial issue hindering the progression of this field is the limitations of RNA-DNA binding interaction technology and the lack of an ncRNAs-DNA-protein international database. Addressing these issues would significantly enhance our understanding of mechanisms that dictate ncRNAs association with myogenesis.

Furthermore, the identification of functional ncRNAs to improve muscle development remains a challenge that needs to be resolved. A major hindrance for most applications is the tissue delivery and distribution of ncRNAs for efficacy (Kaemmerer, 2018). For example, miRNAs function by integrating with complexes (RISC), which impedes their crossing of membranes; lncRNAs have low conservation between species, and their length and higher structure are complex. Currently, the most commonly used gene delivery methods for RNA-based therapeutics are recombinant viral systems such as adenovirus, lentivirus, and adeno-associated viruses (AAVs), which are employed either to inhibit or overexpress miRNAs, circRNAs, and lncRNAs (Sweta et al., 2019). Furthermore, the potential toxicity of the viral vectors should be determined to reduce negative impacts on the receptor. In addition to the delivery system for viral vectors, gene editing technology, antisense oligonucleotides (ASOs), and RNA interference (RNAi)-mediated approaches have also been reported. In recent years, the CRISPR/Cas9 system has been used as an efficient gene editing tool to investigate the function of ncRNAs. However, gene editing technology has presented off-target effects and ethical disputes. In contrast, the ASO technology has fewer off-target effects than the small RNA-mediated approach and the CRISPR/Cas9 system (Bennett and Swayze, 2010). To realize the full potential of ncRNAs as biomarkers or therapeutic targets against muscle, validation studies are warranted using both *in vitro* and *in vivo* systems to illustrate the integrated network of myogenesis.

## Author Contributions

HL and WL wrote the manuscript and selected the literature. BZ and ZX proposed the topic, wrote the manuscript, and corrected and gave suggestions to improve the manuscript. HL, WL, QT, JJ, ZX, and BZ reviewed the manuscript and prepared tables and figures. All authors read and approved the final manuscript.

## Conflict of Interest

The authors declare that the research was conducted in the absence of any commercial or financial relationships that could be construed as a potential conflict of interest.
