# Supplementary material for: Functional Non-coding RNA During Embryonic Myogenesis and Postnatal Muscle Development and Disease
Source: Front Cell Dev Biol. 2021 Jan 28;9:628339. doi: 10.3389/fcell.2021.628339 (PMC7876409; doi:10.3389/fcell.2021.628339)
Supplement: Supplementary file 1 [file Table_1.pdf]

Supplementary Information for

Functional Non-coding RNA During Embryonic Myogenesis and Postnatal Muscle Development and Disease

Hongmei Luo[1,2†], Wei Lv [1,2†], Qian Tong[1,2], Jianjun Jin[1,2], Zaiyan Xu[1,3*] and Bo Zuo[1,2,4*]

*Correspondence:

Zaiyan Xu

xuzaiyan@mail.hzau.edu.cn

Bo Zuo

zuobo@mail.hzau.edu.cn

[†]These authors have contributed equally to this work.

Supplementary Table 1

TABLE 1 miRNAs Target the Same Transcriptional Networks in Different Species

miRNA	Same transcriptional networks	Functions	Species	Reference
miR-1	Targets HDAC4	Promotes proliferation and differentiation	Mouse	(Chen et al., 2006)
		Maintains cell quiescence and promotes satellite cell self-renewal	Goat	(Sui et al., 2019)
miR-27b	Targets Pax3	Promotes differentiation	Mouse	(Crist et al., 2009)
		Promotes proliferation and differentiation	Goat	(Ling et al., 2018)
miR-206	Targets Pax7	Promotes proliferation and differentiation	Mouse	(Chen et al., 2006)
miR-486		Promotes differentiation	Mouse	(Dey et al., 2011)
miR-26a	Targets Smad4	Promotes differentiation	Mouse	(Dey et al., 2012)
miR-675-3p/-5p		Promotes differentiation and regeneration	Mouse	(Dey et al., 2014)
miR-146b		Promotes myogenic differentiation	Mouse	(Khanna et al., 2014)
miR-431		Promotes differentiation and regeneration of old skeletal muscle	Mouse; human	(Lee et al., 2015)

References

Chen, J.F., Mandel, E.M., Thomson, J.M., Wu, Q., Callis, T.E., Hammond, S.M., et al. (2006). The role of microRNA-1 and microRNA-133 in skeletal muscle proliferation and differentiation. *Nat Genet* 38(2)**,** 228-233. doi: 10.1038/ng1725.

Crist, C.G., Montarras, D., Pallafacchina, G., Rocancourt, D., Cumano, A., Conway, S.J., et al. (2009). Muscle stem cell behavior is modified by microRNA-27 regulation of Pax3 expression. *Proc Natl Acad Sci U S A* 106(32)**,** 13383-13387. doi: 10.1073/pnas.0900210106.

Dey, B.K., Gagan, J., and Dutta, A. (2011). miR-206 and -486 induce myoblast differentiation by downregulating Pax7. *Mol Cell Biol* 31(1)**,** 203-214. doi: 10.1128/MCB.01009-10.

Dey, B.K., Gagan, J., Yan, Z., and Dutta, A. (2012). miR-26a is required for skeletal muscle differentiation and regeneration in mice. *Genes Dev* 26(19)**,** 2180-2191. doi: 10.1101/gad.198085.112.

Dey, B.K., Pfeifer, K., and Dutta, A. (2014). The H19 long noncoding RNA gives rise to microRNAs miR-675-3p and miR-675-5p to promote skeletal muscle differentiation and regeneration. *Genes Dev* 28(5)**,** 491-501. doi: 10.1101/gad.234419.113.

Khanna, N., Ge, Y., and Chen, J. (2014). MicroRNA-146b promotes myogenic differentiation and modulates multiple gene targets in muscle cells. *PLoS One* 9(6)**,** e100657. doi: 10.1371/journal.pone.0100657.

Lee, K.P., Shin, Y.J., Panda, A.C., Abdelmohsen, K., Kim, J.Y., Lee, S.M., et al. (2015). miR-431 promotes differentiation and regeneration of old skeletal muscle by targeting Smad4. *Genes Dev* 29(15)**,** 1605-1617. doi: 10.1101/gad.263574.115.

Ling, Y.H., Sui, M.H., Zheng, Q., Wang, K.Y., Wu, H., Li, W.Y., et al. (2018). miR-27b regulates myogenic proliferation and differentiation by targeting Pax3 in goat. *Sci Rep* 8(1)**,** 3909. doi: 10.1038/s41598-018-22262-4.

Sui, Y., Han, Y., Zhao, X., Li, D., and Li, G. (2019). Long non-coding RNA Irm enhances myogenic differentiation by interacting with MEF2D. *Cell Death Dis* 10(3)**,** 181. doi: 10.1038/s41419-019-1399-2.
