# Supplementary material for: Functional Non-coding RNA During Embryonic Myogenesis and Postnatal Muscle Development and Disease
Source: Front Cell Dev Biol. 2021 Jan 28;9:628339. doi: 10.3389/fcell.2021.628339 (PMC7876409; doi:10.3389/fcell.2021.628339)
Supplement: Supplementary file 2 [file Table_2.pdf]

Supplementary Information for

Functional Non-coding RNA During Embryonic Myogenesis and Postnatal Muscle Development and Disease

Hongmei Luo[1,2†], Wei Lv [1,2†], Qian Tong[1,2], Jianjun Jin[1,2], Zaiyan Xu[1,3*] and Bo Zuo[1,2,4*]

*Correspondence:

Zaiyan Xu

xuzaiyan@mail.hzau.edu.cn

Bo Zuo

zuobo@mail.hzau.edu.cn

[†]These authors have contributed equally to this work.

Supplementary Table 2

Table 2 Functional and regulatory mechanisms of lncRNAs in skeletal muscle development

lncRNA	Function	Mechanisms	Subcellular location	Species	Reference
SRA	Promotes differentiation	Coactives MyoD together with the RNA elicase p68/p72	Nucleus	Mouse	(Caretti et al., 2006; Hube et al., 2011)
Gtl2	Promotes skeletal development during embryogenesis	As a PRC2 cofactor that directs PRC2 to the reciprocally imprinted Dlk1 coding gene	Nucleus	Mouse	(Zhao et al., 2010; Zhou et al., 2010)
linc-MD1	Promotes differentiation	Sponges miR-133 and miR-135 to regulate the expression of MAML1 and MEF2C	Cytoplasm	Mouse	(Cesana et al., 2011; Legnini et al., 2014)
lincRNA Yam-1	Inhibits myoblast differentiation	*in cis* regulation of miR-715, which targets Wnt7b	Nucleus/ Cytoplasm	Mouse	(Lu et al., 2013)
m1/2sbs-RNAs	Regulates myogenesis	Binds to the 3' UTR of mRNA, the m1⁄2-sbsRNA–TRAF6 mRNA duplex binds to STAU1, thus degradation of TRAF6 mRNA	Cytoplasm	Mouse	(Wang et al., 2013)
H19	H19 depletion causes accelerated muscle differentiation	Modulates let-7 availability by acting as a molecular sponge	Nucleus/ Cytoplasm	Human	(Kallen et al., 2013)

	Promotes skeletal muscle differentiation and regeneration	Gives rise to miR-675-3p and miR-675-5p to downregulate Smad1, Smad5 and Cdc6	Cytoplasm	Mouse	(Dey et al., 2014)
	Promotes differentiation	As a molecular scaffold facilitates effective association of KSRP with myogenin and other labile transcripts	Cytoplasm	Mouse	(Giovarelli et al., 2014)
	Promotes porcine satellite cell differentiation	Acts as a scaffold to recruit TDP43 to the promoters of MYOD and activates the transcription of MYOD	Nucleus/ Cytoplasm	Porcine	(Li et al., 2020a)
linc-YY1	Promotes differentiation and regeneration	Activates gene expression in trans by interacting with YY1 and removing YY1/PRC2 complex from target promoters	Nucleus	Mouse	(Zhou et al., 2015)
Dum	Promotes differentiation and regeneration	Silence neighboring gene Dppa2 *in cis* by recruiting Dnmts	Nucleus/ Cytoplasm	Mouse	(Wang et al., 2015)
MUNC	Promotes differentiation	Acts *in trans* to promotes gene expression of MyoD, MyoG and Myh3 mRNA; acts as an eRNA for MyoD	Nucleus	Mouse	(Mueller et al., 2015; Cichewicz et al., 2018)
lncMyoD	Promotes differentiation	Regulates the translation of N-Ras and c-Myc by sequestering IMP2 protein	Nucleus/ Cytoplasm	Mouse	(Gong et al., 2015)
lnc-31	Promotes proliferation, inhibits differentiation	Promotes ROCK1 translation by stabilizing YB-1 protein	Nucleus/ Cytoplasm	Mouse	(Ballarino et al., 2015; Dimartino et al., 2018)
Myoregulin (MLN)	Decreases muscle relaxation with reduced exercise performance and Ca2+ uptake into the SR	Binds to SERCA and inhibits its activity	SR/ER membrane	Mouse	(Anderson et al., 2015)

DWORF	Improves muscle contraction capacity and enhances SR Ca2+ uptake	Binds to SERCA and increases its activity	SR membrane	Vertebrates	(Nelson et al., 2016)
Sirt1 AS lncRNA	Promotes proliferation, inhibits differentiation	miR-34a molecular sponge, stabilizes Sirt1 mRNA	Nucleus/ Cytoplasm	Mouse	(Wang et al., 2016)
lnc-MD	Promotes muscle differentiation	Sequesters miR-125b to increase IGF2 expression	Nucleus/ Cytoplasm	Bovine	(Sun et al., 2016)
Malat1	Inhibits differentiation and regeneration	Recruits Suv39h1 to MyoD-binding loci, causing trimethylation of histone 3 lysine 9 (H3K9me3)	Nucleus	Mouse	(Chen et al., 2017)
linc-RAM	Promotes muscle growth and regeneration	Interacts with MyoD and supporting the assembly of MyoD-Baf60c-Brg complex	Nucleus/ Cytoplasm	Mouse	(Yu et al., 2017)
lnc-mg	Promotes differentiation and regeneration	As a ceRNA for miR-125b to control the protein level of IGF2; as a molecular sponge for miR-351-5p, negatively regulating LACTB	Nucleus/ Cytoplasm	Mouse	(Zhu et al., 2017; Du et al., 2019)
lncRNA Gm7325 (myomixer/Minion/ myomerger)	Promotes fusion and regeneration and muscle formation during embryogenesis	Interacts with Myomaker	Membrane	Vertebrates invertebrates	(Quinn et al., 2017; Zhang et al., 2017; Bi et al., 2018; Leikina et al., 2018)
lncRNA *Six1*	Promotes proliferation and muscle growth	Regulates its neighboring gene Six1 *in cis*	Nucleus/ Cytoplasm	Chicken	(Cai et al., 2017)
LINC00961/ 5430416O09Rik (SPAR)	Inhibits muscle regeneration	Interacts with the lysosomal v-ATPase	Endosome/ Lysosome	Mouse and human	(Matsumoto et al., 2017; Tajbakhsh, 2017)
SYISL	Promotes proliferation, inhibits differentiation and muscle regeneration	Inhibits muscle-specific transcription factors through an EZH2-recruitment mechanism	Nucleus/ Cytoplasm	Mouse	(Jin et al., 2018)

Myolinc	Promotes differentiation and regeneration	Recruits TDP-43 to promote of Filip1 in cis, and the Myolinc and TDP-43 complex binds to the promoters of muscle marker genes	Nucleus	Mouse	(Militello et al., 2018)
MAR1	Promotes muscle differentiation and regeneration	ceRNA for miR-487b to regulate Wnt5a protein	Cytoplasm	Mouse	(Zhang et al., 2018b)
linc-smad7	Inhibits proliferation, promotes differentiation and regeneration	miR-125b molecular sponge regulates smad7 and IGF2	Nucleus/ Cytoplasm	Mouse	(Song et al., 2018)
AK017368	Promotes proliferation and suppresses differentiation	ceRNA for miR-30c to regulate trinucleotide repeat containing-6A	Nucleus/ Cytoplasm	Mouse	(Liang et al., 2018)
MUMA	Promotes differentiation	miR-672 molecular sponge	Not determined	Mouse	(Zhang et al., 2018a)
MDNCR	Promotes differentiation	Binds of miR-133a by targeting GosB	Not determined	Bovine	(Li et al., 2018)
Charme	Regulates myogenesis	Acts as a chromatin architect to promote myoblasts differentiation	Nucleus	Mouse and human	(Ballarino et al., 2018)
Neat1	Promotes proliferation and regeneration, inhibits differentiation	Recruits EZH2 to muscle-specific promoters	Nucleus	Mouse	(Wang et al., 2019)
Myoparr	Inhibits proliferation, promotes differentiation	Interacts with MyoD coactivator Ddx17	Nucleus	Mouse	(Hitachi et al., 2019)
Irm	Promotes differentiation and regeneration	Binds to MEF2D and promoting the assembly of MyoD/MEF2D	Nucleus	Mouse	(Sui et al., 2019)
MEG3	Promotes bovine myoblast differentiation	Acts as a ceRNA for miR135 and affects the target gene MEF2C	Nucleus/ Cytoplasm	Bovine	(Liu et al., 2019)

	Promotes differentiation of porcine satellite cells	Spongs miR-423-5p to relieve inhibiting effect on SRF	Cytoplasm	Porcine	(Cheng et al., 2020)
lncIRS1	Promotes proliferation and differentiation	miR-15 molecular sponge	Nucleus/ Cytoplasm	Chicken	(Li et al., 2019)
SAM	Promotes proliferation and regeneration	Interacts and stabilizes Sugt1 thus facilitates Sugt1 mediated kinetochore assembly during cell division	Nucleus	Mouse	(Li et al., 2020c)
MyolncR 4 (1500011 K16RIK)	Promotes muscle formation and regeneration	Codes a 56-aa micropeptide, which was named as LEMP (lncRNA encoded micropeptide)	Plasma membrane and mitochondri a	Mouse and zebrafish	(Wang et al., 2020)
2310043L 19Rik (lnc-231)	Inhibits differentiation and promotes proliferation	As ceRNA to target miR-125a-5p, whereas miR-125a-5p binds to the 3'-UTR of E2F3 mRNA to inhibit its function	Cytoplasm	Mouse	(Li et al., 2020b)
SMaRT	Regulates early myogenesis	Controls translation of a G-quadruplex-containing mRNA antagonizing the DHX36 helicase	Cytoplasm	Mouse	(Martone et al., 2020)
Ppp1r1b-lncRNA	Promotes myogenic differentiation	Compets for PRC2 binding with chromatin of myogenic master regulators	Nucleus	Mouse and human	(Martone et al., 2020)
MGPF	Promotes myogenic differentiation	miR-135a-5p molecular sponge; enhances HuR-mediated mRNA stabilization of MRFs	Nucleus/ Cytoplasm	Mouse, porcine and human	(Lv et al., 2020)

References

Anderson, D.M., Anderson, K.M., Chang, C.L., Makarewich, C.A., Nelson, B.R., McAnally, J.R., et al. (2015). A micropeptide encoded by a putative long noncoding RNA regulates muscle performance. *Cell* 160(4), 595-606. doi: 10.1016/j.cell.2015.01.009.

Ballarino, M., Cazzella, V., D'Andrea, D., Grassi, L., Bisceglie, L., Cipriano, A., et al. (2015). Novel long noncoding RNAs (lncRNAs) in myogenesis: a miR-31 overlapping lncRNA transcript controls myoblast differentiation. *Mol Cell Biol* 35(4), 728-736. doi: 10.1128/MCB.01394-14.
Ballarino, M., Cipriano, A., Tita, R., Santini, T., Desideri, F., Morlando, M., et al. (2018). Deficiency in the nuclear long noncoding RNA Charme causes myogenic defects and heart remodeling in mice. *EMBO J* 37(18). doi: 10.15252/embj.201899697.
Bi, P., McAnally, J.R., Shelton, J.M., Sanchez-Ortiz, E., Bassel-Duby, R., and Olson, E.N. (2018). Fusogenic micropeptide Myomixer is essential for satellite cell fusion and muscle regeneration. *Proc Natl Acad Sci U S A* 115(15), 3864-3869. doi: 10.1073/pnas.1800052115.
Cai, B., Li, Z., Ma, M., Wang, Z., Han, P., Abdalla, B.A., et al. (2017). LncRNA-Six1 Encodes a Micropeptide to Activate Six1 in Cis and Is Involved in Cell Proliferation and Muscle Growth. *Front Physiol* 8, 230. doi: 10.3389/fphys.2017.00230.
Caretti, G., Schiltz, R.L., Dilworth, F.J., Di Padova, M., Zhao, P., Ogryzko, V., et al. (2006). The RNA helicases p68/p72 and the noncoding RNA SRA are coregulators of MyoD and skeletal muscle differentiation. *Dev Cell* 11(4), 547-560. doi: 10.1016/j.devcel.2006.08.003.
Cesana, M., Cacchiarelli, D., Legnini, I., Santini, T., Sthandier, O., Chinappi, M., et al. (2011). A long noncoding RNA controls muscle differentiation by functioning as a competing endogenous RNA. *Cell* 147(2), 358-369. doi: 10.1016/j.cell.2011.09.028.
Chen, X., He, L., Zhao, Y., Li, Y., Zhang, S., Sun, K., et al. (2017). Malat1 regulates myogenic differentiation and muscle regeneration through modulating MyoD transcriptional activity. *Cell Discov* 3, 17002. doi: 10.1038/celldisc.2017.2.
Cheng, X., Li, L., Shi, G., Chen, L., Fang, C., Li, M., et al. (2020). MEG3 Promotes Differentiation of Porcine Satellite Cells by Sponging miR-423-5p to Relieve Inhibiting Effect on SRF. *Cells* 9(2). doi: 10.3390/cells9020449.
Cichewicz, M.A., Kiran, M., Przanowska, R.K., Sobierajska, E., Shibata, Y., and Dutta, A. (2018). MUNC, an Enhancer RNA Upstream from the MYOD Gene, Induces a Subgroup of Myogenic Transcripts in trans Independently of MyoD. *Mol Cell Biol* 38(20). doi: 10.1128/MCB.00655-17.
Dey, B.K., Pfeifer, K., and Dutta, A. (2014). The H19 long noncoding RNA gives rise to microRNAs miR-675-3p and miR-675-5p to promote skeletal muscle differentiation and regeneration. *Genes Dev* 28(5), 491-501. doi: 10.1101/gad.234419.113.
Dimartino, D., Colantoni, A., Ballarino, M., Martone, J., Mariani, D., Danner, J., et al. (2018). The Long Non-coding RNA lnc-31 Interacts with Rock1 mRNA and Mediates Its YB-1-Dependent Translation. *Cell Rep* 23(3), 733-740. doi: 10.1016/j.celrep.2018.03.101.
Du, J., Zhang, P., Zhao, X., He, J., Xu, Y., Zou, Q., et al. (2019). MicroRNA-351-5p mediates skeletal myogenesis by directly targeting lactamase-beta and is regulated by lnc-mg. *FASEB J* 33(2), 1911-1926. doi: 10.1096/fj.201701394RRR.
Giovarelli, M., Bucci, G., Ramos, A., Bordo, D., Wilusz, C.J., Chen, C.Y., et al. (2014). H19 long noncoding RNA controls the mRNA decay promoting function of KSRP. *Proc Natl Acad Sci U S A* 111(47), E5023-5028. doi: 10.1073/pnas.1415098111.

Gong, C., Li, Z., Ramanujan, K., Clay, I., Zhang, Y., Lemire-Brachat, S., et al. (2015). A long non-coding RNA, LncMyoD, regulates skeletal muscle differentiation by blocking IMP2-mediated mRNA translation. *Dev Cell* 34(2), 181-191. doi: 10.1016/j.devcel.2015.05.009.

Hitachi, K., Nakatani, M., Takasaki, A., Ouchi, Y., Uezumi, A., Ageta, H., et al. (2019). Myogenin promoter-associated lncRNA Myoparr is essential for myogenic differentiation. *EMBO Rep* 20(3). doi: 10.15252/embr.201847468.

Hube, F., Velasco, G., Rollin, J., Furling, D., and Francastel, C. (2011). Steroid receptor RNA activator protein binds to and counteracts SRA RNA-mediated activation of MyoD and muscle differentiation. *Nucleic Acids Res* 39(2), 513-525. doi: 10.1093/nar/gkq833.

Jin, J.J., Lv, W., Xia, P., Xu, Z.Y., Zheng, A.D., Wang, X.J., et al. (2018). Long noncoding RNA SYISL regulates myogenesis by interacting with polycomb repressive complex 2. *Proc Natl Acad Sci U S A* 115(42), E9802-E9811. doi: 10.1073/pnas.1801471115.

Kallen, A.N., Zhou, X.B., Xu, J., Qiao, C., Ma, J., Yan, L., et al. (2013). The imprinted H19 lncRNA antagonizes let-7 microRNAs. *Mol Cell* 52(1), 101-112. doi: 10.1016/j.molcel.2013.08.027.

Legnini, I., Morlando, M., Mangiavacchi, A., Fatica, A., and Bozzoni, I. (2014). A feedforward regulatory loop between HuR and the long noncoding RNA linc-MD1 controls early phases of myogenesis. *Mol Cell* 53(3), 506-514. doi: 10.1016/j.molcel.2013.12.012.

Leikina, E., Gamage, D.G., Prasad, V., Goykhberg, J., Crowe, M., Diao, J., et al. (2018). Myomaker and Myomerger Work Independently to Control Distinct Steps of Membrane Remodeling during Myoblast Fusion. *Dev Cell* 46(6), 767-780 e767. doi: 10.1016/j.devcel.2018.08.006.

Li, H., Yang, J., Jiang, R., Wei, X., Song, C., Huang, Y., et al. (2018). Long Non-coding RNA Profiling Reveals an Abundant MDNCR that Promotes Differentiation of Myoblasts by Sponging miR-133a. *Mol Ther Nucleic Acids* 12, 610-625. doi: 10.1016/j.omtn.2018.07.003.

Li, J., Zhao, W., Li, Q., Huang, Z., Shi, G., and Li, C. (2020a). Long Non-Coding RNA H19 Promotes Porcine Satellite Cell Differentiation by Interacting with TDP43. *Genes (Basel)* 11(3). doi: 10.3390/genes11030259.

Li, R., Li, B., Shen, M., Cao, Y., Zhang, X., Li, W., et al. (2020b). LncRNA 2310043L19Rik inhibits differentiation and promotes proliferation of myoblast by sponging miR-125a-5p. *Aging (Albany NY)* 12(7), 5625-5639. doi: 10.18632/aging.102905.

Li, Y., Yuan, J., Chen, F., Zhang, S., Zhao, Y., Chen, X., et al. (2020c). Long noncoding RNA SAM promotes myoblast proliferation through stabilizing Sugt1 and facilitating kinetochore assembly. *Nat Commun* 11(1), 2725. doi: 10.1038/s41467-020-16553-6.

Li, Z., Cai, B., Abdalla, B.A., Zhu, X., Zheng, M., Han, P., et al. (2019). LncIRS1 controls muscle atrophy via sponging miR-15 family to activate IGF1-PI3K/AKT pathway. *J Cachexia Sarcopenia Muscle* 10(2), 391-410. doi: 10.1002/jcsm.12374.

Liang, T., Zhou, B., Shi, L., Wang, H., Chu, Q., Xu, F., et al. (2018). lncRNA AK017368 promotes proliferation and suppresses differentiation of myoblasts in skeletal muscle development by attenuating the function of miR-30c. *FASEB J* 32(1), 377-389. doi: 10.1096/fj.201700560RR.

Liu, M., Li, B., Peng, W., Ma, Y., Huang, Y., Lan, X., et al. (2019). LncRNA-MEG3 promotes bovine myoblast differentiation by sponging miR-135. *J Cell Physiol* 234(10)**,** 18361-18370. doi: 10.1002/jcp.28469.

Lu, L., Sun, K., Chen, X., Zhao, Y., Wang, L., Zhou, L., et al. (2013). Genome-wide survey by ChIP-seq reveals YY1 regulation of lincRNAs in skeletal myogenesis. *EMBO J* 32(19)**,** 2575-2588. doi: 10.1038/emboj.2013.182.

Lv, W., Jin, J., Xu, Z., Luo, H., Guo, Y., Wang, X., et al. (2020). lncMGPF is a novel positive regulator of muscle growth and regeneration. *J Cachexia Sarcopenia Muscle*. doi: 10.1002/jcsm.12623.

Martone, J., Mariani, D., Santini, T., Setti, A., Shamloo, S., Colantoni, A., et al. (2020). SMaRT lncRNA controls translation of a G-quadruplex-containing mRNA antagonizing the DHX36 helicase. *EMBO Rep* 21(6)**,** e49942. doi: 10.15252/embr.201949942.

Matsumoto, A., Pasut, A., Matsumoto, M., Yamashita, R., Fung, J., Monteleone, E., et al. (2017). mTORC1 and muscle regeneration are regulated by the LINC00961-encoded SPAR polypeptide. *Nature* 541(7636)**,** 228-232. doi: 10.1038/nature21034.

Militello, G., Hosen, M.R., Ponomareva, Y., Gellert, P., Weirick, T., John, D., et al. (2018). A novel long non-coding RNA Myolinc regulates myogenesis through TDP-43 and Filip1. *J Mol Cell Biol* 10(2)**,** 102-117. doi: 10.1093/jmcb/mjy025.

Mueller, A.C., Cichewicz, M.A., Dey, B.K., Layer, R., Reon, B.J., Gagan, J.R., et al. (2015). MUNC, a long noncoding RNA that facilitates the function of MyoD in skeletal myogenesis. *Mol Cell Biol* 35(3)**,** 498-513. doi: 10.1128/MCB.01079-14.

Nelson, B.R., Makarewich, C.A., Anderson, D.M., Winders, B.R., Troupes, C.D., Wu, F., et al. (2016). A peptide encoded by a transcript annotated as long noncoding RNA enhances SERCA activity in muscle. *Science* 351(6270)**,** 271-275. doi: 10.1126/science.aad4076.

Quinn, M.E., Goh, Q., Kurosaka, M., Gamage, D.G., Petrany, M.J., Prasad, V., et al. (2017). Myomerger induces fusion of non-fusogenic cells and is required for skeletal muscle development. *Nat Commun* 8**,** 15665. doi: 10.1038/ncomms15665.

Song, C., Wang, J., Ma, Y., Yang, Z., Dong, D., Li, H., et al. (2018). Linc-smad7 promotes myoblast differentiation and muscle regeneration via sponging miR-125b. *Epigenetics* 13(6)**,** 591-604. doi: 10.1080/15592294.2018.1481705.

Sui, Y., Han, Y., Zhao, X., Li, D., and Li, G. (2019). Long non-coding RNA Irm enhances myogenic differentiation by interacting with MEF2D. *Cell Death Dis* 10(3)**,** 181. doi: 10.1038/s41419-019-1399-2.

Sun, X., Li, M., Sun, Y., Cai, H., Lan, X., Huang, Y., et al. (2016). The developmental transcriptome sequencing of bovine skeletal muscle reveals a long noncoding RNA, lncMD, promotes muscle differentiation by sponging miR-125b. *Biochim Biophys Acta* 1863(11)**,** 2835-2845. doi: 10.1016/j.bbamcr.2016.08.014.

Tajbakhsh, S. (2017). lncRNA-Encoded Polypeptide SPAR(s) with mTORC1 to Regulate Skeletal Muscle Regeneration. *Cell Stem Cell* 20(4)**,** 428-430. doi: 10.1016/j.stem.2017.03.016.

Wang, G.Q., Wang, Y., Xiong, Y., Chen, X.C., Ma, M.L., Cai, R., et al. (2016). Sirt1 AS lncRNA interacts with its mRNA to inhibit muscle formation by attenuating function of miR-34a. *Sci Rep* 6**,** 21865. doi: 10.1038/srep21865.

Wang, J., Gong, C., and Maquat, L.E. (2013). Control of myogenesis by rodent SINE-containing lncRNAs. *Genes Dev* 27(7)**,** 793-804. doi: 10.1101/gad.212639.112.

Wang, L., Fan, J., Han, L., Qi, H., Wang, Y., Wang, H., et al. (2020). The micropeptide LEMP plays an evolutionarily conserved role in myogenesis. *Cell Death Dis* 11(5)**,** 357. doi: 10.1038/s41419-020-2570-5.

Wang, L., Zhao, Y., Bao, X., Zhu, X., Kwok, Y.K., Sun, K., et al. (2015). LncRNA Dum interacts with Dnmts to regulate Dppa2 expression during myogenic differentiation and muscle regeneration. *Cell Res* 25(3)**,** 335-350. doi: 10.1038/cr.2015.21.

Wang, S., Zuo, H., Jin, J., Lv, W., Xu, Z., Fan, Y., et al. (2019). Long noncoding RNA Neat1 modulates myogenesis by recruiting Ezh2. *Cell Death Dis* 10(7)**,** 505. doi: 10.1038/s41419-019-1742-7.

Yu, X., Zhang, Y., Li, T., Ma, Z., Jia, H., Chen, Q., et al. (2017). Long non-coding RNA Linc-RAM enhances myogenic differentiation by interacting with MyoD. *Nat Commun* 8**,** 14016. doi: 10.1038/ncomms14016.

Zhang, Q., Vashisht, A.A., O'Rourke, J., Corbel, S.Y., Moran, R., Romero, A., et al. (2017). The microprotein Minion controls cell fusion and muscle formation. *Nat Commun* 8**,** 15664. doi: 10.1038/ncomms15664.

Zhang, Z.K., Li, J., Guan, D., Liang, C., Zhuo, Z., Liu, J., et al. (2018a). Long Noncoding RNA lncMUMA Reverses Established Skeletal Muscle Atrophy following Mechanical Unloading. *Mol Ther* 26(11)**,** 2669-2680. doi: 10.1016/j.ymthe.2018.09.014.

Zhang, Z.K., Li, J., Guan, D., Liang, C., Zhuo, Z., Liu, J., et al. (2018b). A newly identified lncRNA MAR1 acts as a miR-487b sponge to promote skeletal muscle differentiation and regeneration. *J Cachexia Sarcopenia Muscle* 9(3)**,** 613-626. doi: 10.1002/jcsm.12281.

Zhao, J., Ohsumi, T.K., Kung, J.T., Ogawa, Y., Grau, D.J., Sarma, K., et al. (2010). Genome-wide identification of polycomb-associated RNAs by RIP-seq. *Mol Cell* 40(6)**,** 939-953. doi: 10.1016/j.molcel.2010.12.011.

Zhou, L., Sun, K., Zhao, Y., Zhang, S., Wang, X., Li, Y., et al. (2015). Linc-YY1 promotes myogenic differentiation and muscle regeneration through an interaction with the transcription factor YY1. *Nat Commun* 6**,** 10026. doi: 10.1038/ncomms10026.

Zhou, Y., Cheunsuchon, P., Nakayama, Y., Lawlor, M.W., Zhong, Y., Rice, K.A., et al. (2010). Activation of paternally expressed genes and perinatal death caused by deletion of the Gtl2 gene. *Development* 137(16)**,** 2643-2652. doi: 10.1242/dev.045724.

Zhu, M., Liu, J., Xiao, J., Yang, L., Cai, M., Shen, H., et al. (2017). Lnc-mg is a long non-coding RNA that promotes myogenesis. *Nat Commun* 8**,** 14718. doi: 10.1038/ncomms14718.
